# Case Report: Low-grade glioma with *NF1* loss of function mimicking diffuse intrinsic pontine glioma

**DOI:** 10.3389/fsurg.2024.1356660

**Published:** 2024-05-22

**Authors:** Joshua D. Bernstock, Paramesh V. Karandikar, Jason A. Chen, Jakob Seidlitz, Gregory K. Friedman, David M. Meredith, Kevin X. Liu, Daphne Haas-Kogan, David A. Reardon, Pier Paolo Peruzzi

**Affiliations:** ^1^ Department of Neurosurgery, Brigham and Women’s Hospital, Boston, MA, United States; ^2^ Department of Neurosurgery, Boston Children’s Hospital, Boston, MA, United States; ^3^ Department of Child and Adolescent Psychiatry and Behavioral Sciences, The Children’s Hospital of Philadelphia, Philadelphia, PA, United States; ^4^ Department of Psychiatry, University of Pennsylvania, Philadelphia, PA, United States; ^5^ Lifespan Brain Institute, The Children’s Hospital of Philadelphia and Penn Medicine, Philadelphia, PA, United States; ^6^ Division of Pediatrics, Neuro-Oncology Section, The University of Texas MD Anderson Cancer Center, Houston, TX, United States; ^7^ Department of Pathology, Brigham and Women’s Hospital, Boston, MA, United States; ^8^ Department of Radiation Oncology, Brigham and Women’s Hospital, Dana-Farber Cancer Institute, Boston Children’s Hospital, Boston, MA, United States; ^9^ Department of Medical Oncology, Center for Neuro-Oncology, Dana-Farber Cancer Institute, Brigham and Women’s Hospital, Boston, MA, United States

**Keywords:** DIPG, pontine glioma, *NF1*, biopsy-based diagnosis, glioma

## Abstract

Intrinsic, expansile pontine tumors typically occur in the pediatric population. These tumors characteristically present as diffuse intrinsic pontine glioma (DIPG), which is now considered as diffuse midline glioma (DMG), H3K27-mutated of the pons. DIPG has limited treatment options and a poor prognosis, and the value of tissue diagnosis from an invasive biopsy remains controversial. This study presents the case of a 19-year-old female with clinical and imaging hallmarks of DIPG, who underwent a biopsy of a tumor in the region of the right middle cerebellar peduncle. Her lesional cells were negative for H3K27M alterations and had low-grade histologic features. Next-generation sequencing revealed a frameshift mutation in the *NF1* gene as the likely driver mutation. These features suggest a diagnosis of a low-grade glioma associated with *NF1* loss of function, with far-reaching consequences regarding both treatment strategy and prognosis. This case provides support for the utility of diagnostic tissue biopsy in cases of suspected DIPG.

## 1 Introduction

Gliomas represent a broad subset of neoplastic diseases within the central nervous system (CNS). Their presentations and prognoses vary based on factors such as precursor cell type, anatomical location, and molecular characteristics (1). Low-grade gliomas have longer survival periods (2) compared with diffuse midline gliomas (DMGs), including diffuse intrinsic pontine gliomas (DIPGs), which are expansile pontine tumors that are usually present in pediatric populations (1, 3). As such, the management principles for glioma widely vary depending on the characteristics of the tumor, ranging from surveillance for low-grade disease progression to palliation for highly aggressive manifestations.

While the diagnosis and classification of gliomas had previously been performed based on anatomic locale, clinical features, and histopathology, the 2021 revision of the World Health Organization's (WHO) classification for CNS tumors has significantly featured molecular characteristics as a means of definitive diagnosis (1). The increasing prevalence of molecular diagnostic techniques in the clinical setting, coupled with targeted therapeutics, makes molecular diagnosis a powerful tool for identifying the most effective treatments for a given tumor. For example, DIPG is now considered a subset of DMG, with H3K27 alteration, characterized by the nominal histone mutation that portends a poor prognosis (1). The management of DIPG typically entails radiation therapy to the pons as its diffuse and aggressive nature precludes surgical resection. Therefore, treatment often proceeds based on characteristic imaging features alone. Due to the risk associated with performing a biopsy on the eloquent tissue of the brainstem, biopsies have traditionally been avoided, although they are being utilized more frequently as researchers attempt to better understand the disease and develop effective therapies for it (4).

This study presents the case of a 19-year-old female who presented with migraine headache, gait instability, and lower extremity paresthesia with brain imaging suggestive of DIPG. However, the tissue diagnosis revealed bland histopathological features associated with a single mutation in the *NF1* gene. These findings crucially altered the treatment paradigm following the biopsy.

## 2 Case description

**Figure 1:**
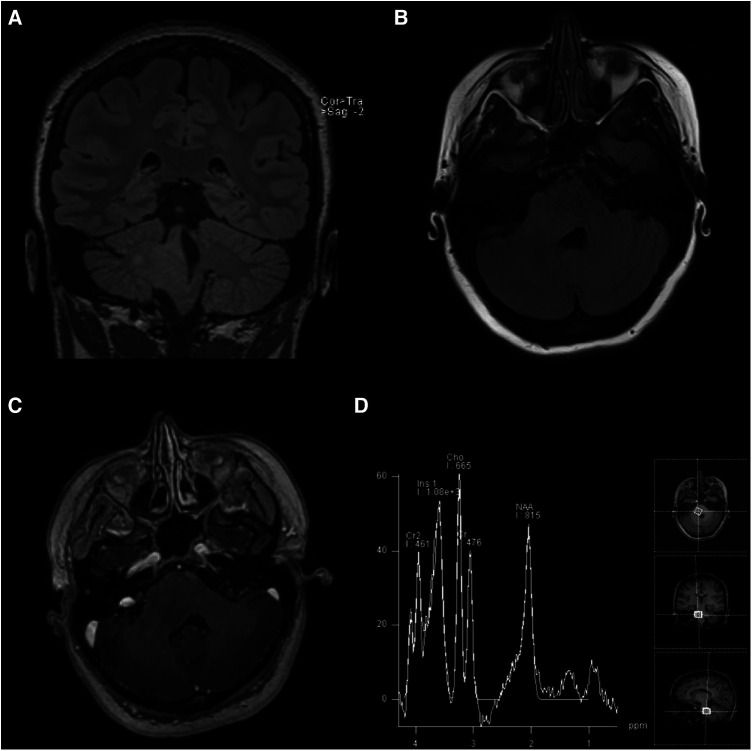
MRI scans taken on initial admission: (**A**) coronal T2-weighted FLAIR, (**B**) axial T2-weighted FLAIR, (**C**) axial post-gadolinium contrast T1-weighted, and (**D**) magnetic resonance spectroscopy sequences demonstrating the non-enhancing, expansile right pontine lesion with an increased choline peak suggestive of infiltrating glioma.

A 19-year-old female with a multi-year history of ocular migraines presented to the emergency department with ataxia and lower extremity paresthesia preceded by refractory holocranial migraine. The patient noted that the headaches differed from her previous migraines and had been increasing in severity and duration over the preceding 8 months, culminating in new-onset dizziness, gait instability, and numbness and tingling in her legs over the preceding weeks (Table 1). She was scheduled for neuroimaging because of the progression of symptoms. Magnetic resonance imaging (MRI) revealed an ill-defined mass within the right pons that extended into the right middle cerebellar peduncle and the right posterolateral medulla with a corresponding partial effacement of the fourth ventricle suggestive of DIPG (Figure 1). The supratentorial gray and white matter volumes were within the normal volumetric percentile ranges for her age (5), and subsequent MRI spectroscopy revealed a markedly elevated choline level with decreased NAA in the lesion when compared against the left pons and the middle cerebellar peduncle. In another hospital, the biopsy was initially deferred due to the location of the lesion in the brainstem and the presumptive diagnosis of DIPG, with a recommendation to proceed with standard radiotherapy. However, the patient was transferred to the Brigham and Women's Hospital/Dana-Farber Cancer Institute (DFCI) for further care, where the recommendation to perform a biopsy prior to radiotherapy was made to definitively ascertain the histopathologic and molecular genetic features to determine potential eligibility for ongoing clinical trials.

**Table 1:** Clinical timeline.

Time	Event
November 2022	- New-onset migraine headaches
May 2023	- Increased migraine severity and duration - New-onset dizziness, gait instability, and sporadic lower extremity paresthesia
July 2023	- Initial MRI demonstrating a poorly defined right pontine mass extending into the right middle cerebral peduncle and the right posterolateral medulla with the fourth ventricle effacement suggestive of DIPG
August 2023	- Suboccipital craniotomy with open biopsy - Initial immunohistochemistry negative for MIB-1, IDH1, R132H, and H3F3A K27 - Initiated on dexamethasone 4 mg TID, valproate 250 mg XR qd, acetaminophen and Fioricet PRN, and walker
Early October 2023	- Dexamethasone decreased to 2 mg qd - Initiated on gabapentin 600 mg BID
Late October 2023	- Dexamethasone decreased to 1 mg qd - MRI brain demonstrates a stable lesion

## 3 Diagnostic assessment

**Figure 2:**
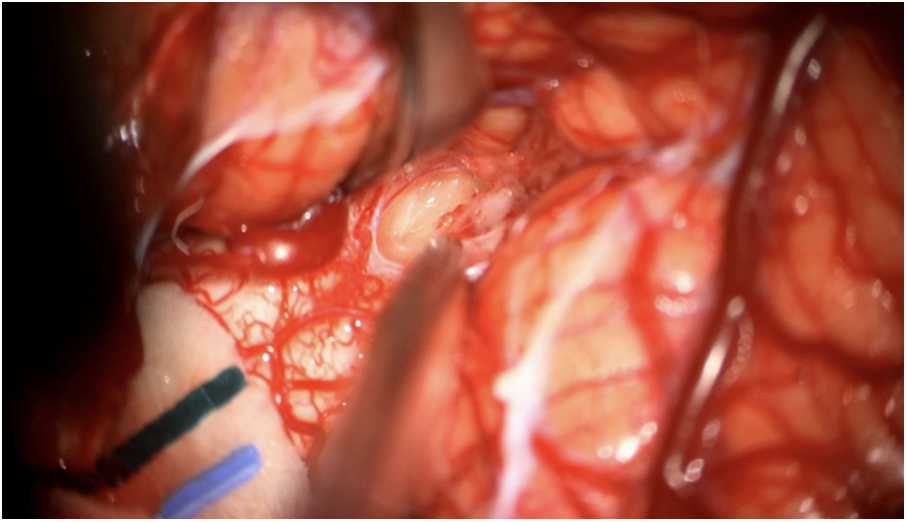
Intraoperative photograph of the mass lesion taken during the biopsy.

**Figure 3:**
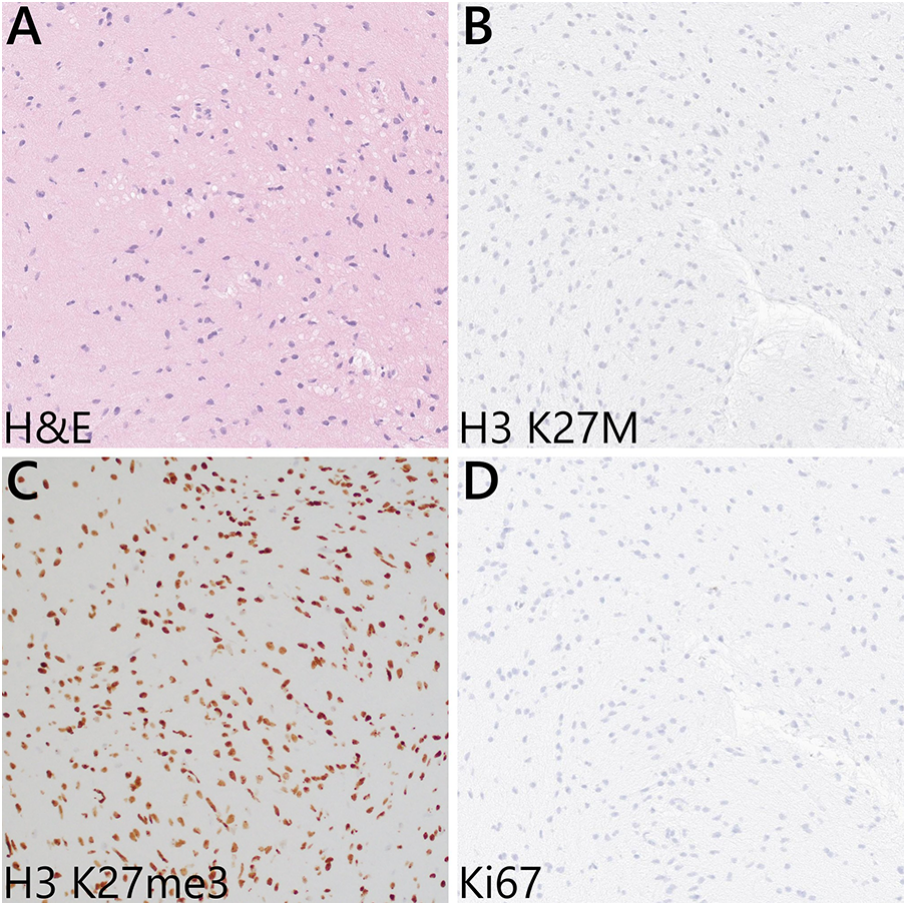
Representative microscopic features from the intraoperative biopsy sample. (**A**) H&E sections showed mildly hypercellular white matter with infiltrating atypical glial cells—notably, Rosenthal fibers, eosinophilic granular bodies, microvascular proliferation, and necrosis are absent. (**B**) Immunohistochemistry for H3 K27M was negative, and (**C**) the H3 K27 trimethylation (me3) expression was retained in the tumor cells. (**D**) Ki67 labeling was negative.

An open biopsy utilizing neuronavigation and intraoperative electrophysiology was decided upon due to the exophytic components of the mass. A posterior approach with craniotomy to widen the foramen magnum and C1 laminectomy was performed, followed by microsurgical dissection of the right tonsil. Regions of discolored tissue were observed on the floor of the fourth ventricle (Figure 2). A monopolar stimulator was used to map adjacent critical neuroanatomy, and micropituitary cup forceps were used to sample regions of abnormal-appearing tissue, which were silent upon stimulation. The intraoperative frozen section analysis was consistent with the glioma, showing minimal hypercellularity and rare atypical cells. Immunohistochemistry with mutation-specific antibodies against IDH1 R132H and H3K27M was negative, while H3 K27 trimethylation expression was retained (Figure 3). The MIB-1 proliferation index was 0%. Notably, targeted next-generation sequencing (Oncopanel, Table 2) revealed a frameshift variant in exon 3 of the *NF1* gene (c.276dup, p.C93Mfs*14) in 36% of the reads with an accompanying loss of 17q heterozygosity. There were no copy number variants, structural variants, or known pathogenic variants in other assayed genes, including *IDH1*, *IDH2*, *H3F3A*, *EGFR*, *FGFR1*, *PDGFRA*, *CDKN2A/B*, and *BRAF*, by sequencing. The lack of mutations associated with DMG, namely, H3 K27M/I or alterations in *TP53*, *ACVR1*, *PDGFRA*, or *EGFR*, retained H3 K27 trimethylation. Low-grade infiltrative histology did not cleanly fit within a current diagnostic entity per the 2021 WHO criteria (1). As a result, the patient was diagnosed with a WHO grade 1 or 2 diffuse low-grade glioma, not otherwise specified.

**Table 2:** Oncopanel findings.

Index	Result
Percentage of neoplastic cells in the specimen	50%
Tumor mutational burden/megabase	2.281 (13th percentile for glioma)
Mismatch repair	Proficient
Mutations	Tier 1: NF1 c.276dup (p.C93Mfs*14), exon 3 Tier 2: none Tier 3: none Tier 4: DNMT3A c.1924G > A (p.G642R), exon 16; SLX 4 c.2779G > A (p.A927T), exon 12
Structural variants	None identified
Copy number variants	None identified
Pertinent negatives	ACVR1 (codon 328) BRAF (codons 464, 466, 469, 581, 594, 597, 600, and 601) EGFR (exons 19 and 20; codons 709, 719, 790, 797, 858, and 861) FGFR1 (codons 546 and 656) H3F3A (codons 27 and 34) HIST1H3B (codon 27) HIST1H3C (codon 27) IDH1 (codon 132) IDH2 (codons 140 and 172) PDGFRA (codon 842) PIK3CA (codons 118, 345, 420,453, 542, 545, and 1047) DAXX MSH6 PIK3C2B PIK3R1 TP53 CDKN2A
Pertinent insufficient coverage	ATRX (exon 1) CIC (exon 1) PTEN (exon 3) RB1 (exons 6 and 14)

The oncopanel findings found on the biopsy specimens were generally bland. Note the absence of mutations associated with DMG (i.e., BRAF, EGFR, H3F3A, HIST1H3B, and IDH 1/2) and adult/pediatric low-grade glioma (adult, IDH1/2; pediatric, FGFR1, BRAF) (6).

On postoperative follow-up, the patient recovered well with gradual improvement in her neurologic status and no new neurologic symptoms. She tapered off dexamethasone. The subsequent work-up for neurofibromatosis indicated that the mutation was somatic. She lacked any stigmata of neurofibromatosis upon examination, and the sequencing of the *NF1* gene from a peripheral blood sample did not reveal any variants. The follow-up MRI at 3 and 5 months post-biopsy demonstrated a stable lesion. Our patient continues to do well clinically with gradual neurologic improvement. She is participating in physical therapy and has resumed her college classes. Her presenting symptoms of headache, ataxia, and lower extremity paresthesia have remained stable.

## 4 Discussion

This study's presentation of low-grade glioma in a region typically associated with DIPG highlights the limitations of neuroimaging in differentiating infiltrative tumors of the CNS and suggests a critical role for molecular diagnosis in such circumstances. In the case of our patient, the mass lesion demonstrated radiographical features consistent with DIPG, particularly effacement of the fourth ventricle, also known as the “flat floor of fourth ventricle sign,” and minimal enhancement on contrast MRI. Although DIPG is the most common form of pontine glioma, the fact that radiographic features are non-specific for DIPG compared to other histopathologic types suggests that imaging characteristics alone may be insufficient for diagnosis (7). The morbidity and mortality associated with brainstem biopsies have been the source of historical apprehension: the most commonly reported complications include cranial nerve palsy (4.2%, in particular, CN VII), perioperative hemorrhage (3.6%), hemiparesis (2.1%), and disorders of speech or movement (2.1 and 1.0%, respectively) (8). However, recent studies suggest that biopsy can safely be done in experienced centers. A meta-analysis by Kickingereder et al. (9), which included 1,480 cases of stereotactic biopsy for brainstem tumors, reported a 96.2% diagnostic success rate at the cost of 1.7% permanent morbidity and 0.9% mortality. A more recent study by Hamisch et al. (10) on 735 cases in the pediatric population reported a 96.1% diagnostic success rate with 0.6% permanent morbidity and 0.6% mortality. The risk of complications secondary to non-indicated therapeutic irradiation must also be considered. In this case, the decision to perform an open biopsy vs. a more common needle biopsy was informed by the dorsally exophitic nature of the lesion, which allowed a direct approach to the tumor with minimal-to-no violation of the adjacent brain.

The importance of molecular diagnosis is further accentuated by the rarity of the genetic profile observed in our patient's tumor. The presence of a putative loss-of-function mutation in the *NF1* gene in a subset of reads and in the absence of any of the clinical features associated with neurofibromatosis type 1 (NF1, also known as von Recklinghausen's disease) suggests *NF1* mosaicism, a rare phenomenon previously reported in patients with a higher degree of *NF1* mutagenic burden in the brain or glioma tissue relative to the somatic tissue (11). The patient did not demonstrate any symptoms of germline *NF1* mutation, and the sequencing of a peripheral blood sample did not demonstrate the loss-of-function mutation. There was no family history of neurofibromatosis. In addition, the patient's tumor lacked the genetic markers that are commonly associated with pediatric or adult low-grade glioma, such as mutations in *IDH1/2*, *FGFR1*, *BRAF*, and histone H3 encoding genes (6). We therefore considered the tumor to be related to somatic mosaicism or somatic mutation leading to a biallelic *NF1* loss of function. Given the low-grade histologic features, low MIB-1 index, and absence of H3 K27M, we concluded that this tumor is most consistent with low-grade gliomas commonly observed in NF1 patients. Fortunately, the prognosis for these patients is much better than that associated with DIPG. The aggregate findings in our case were consistent with an NF-1 mutant, pediatric low-grade glioma; hence, the decision was made to forego radiation therapy until there is clinical or radiographic evidence of tumor growth during surveillance follow-up.

The generalizability of our findings to other cases of suspected DIPG is limited due to the fact that this is a report of a single case with limited follow-up thus far. However, this case is emblematic because of the marked impact the tissue diagnosis has had on this patient's prognosis and management. Notably, other recent studies have also demonstrated that molecular information obtained from biopsies can guide potential treatment options, including the use of targeted inhibitors (12–15). Nonetheless, given the potential for complications and the incredible heterogeneity in the presentation of pontine glioneuronal masses, we suggest that the decision on whether to perform a biopsy should be based on the clinical judgment of risk and benefit by the surgeon and neuro-oncologist as well as shared decision-making with the patient.

In conclusion, the case presented in this study provides further support to the importance of tumor diagnosis through biopsy and detailed histopathologic and molecular genetic profiling of diffuse brainstem tumors.

## Data Availability

The original contributions presented in the study are included in the article/Supplementary Material. Further inquiries can be directed to the corresponding author/s.
